# The effect of stimulation type, head modeling, and combined EEG and MEG on the source reconstruction of the somatosensory P20/N20 component

**DOI:** 10.1002/hbm.24754

**Published:** 2019-08-09

**Authors:** Marios Antonakakis, Sophie Schrader, Andreas Wollbrink, Robert Oostenveld, Stefan Rampp, Jens Haueisen, Carsten H. Wolters

**Affiliations:** ^1^ Institute for Biomagnetism and Biosignalanalysis, University of Muenster Muenster Germany; ^2^ Donders Institute, Radboud University Nijmegen Netherlands; ^3^ Karolinska Institute Stockholm Sweden; ^4^ Department of Neurosurgery University Hospital Erlangen Erlangen Germany; ^5^ Institute for Biomedical Engineering and Informatics, Technical University of Ilmenau Ilmenau Germany; ^6^ Otto Creutzfeldt Center for Cognitive and Behavioral Neuroscience University of Münster Münster Germany

**Keywords:** EEG, finite element method, MEG, multimodal imaging, somatosensory cortex, somatosensory evoked fields, somatosensory evoked potentials

## Abstract

Modeling and experimental parameters influence the Electro‐ (EEG) and Magnetoencephalography (MEG) source analysis of the somatosensory P20/N20 component. In a sensitivity group study, we compare P20/N20 source analysis due to different stimulation type (Electric‐Wrist [EW], Braille‐Tactile [BT], or Pneumato‐Tactile [PT]), measurement modality (combined EEG/MEG – EMEG, EEG, or MEG) and head model (standard or individually skull‐conductivity calibrated including brain anisotropic conductivity). Considerable differences between pairs of stimulation types occurred (EW‐BT: 8.7 ± 3.3 mm/27.1° ± 16.4°, BT‐PT: 9 ± 5 mm/29.9° ± 17.3°, and EW‐PT: 9.8 ± 7.4 mm/15.9° ± 16.5° and 75% strength reduction of BT or PT when compared to EW) regardless of the head model used. EMEG has nearly no localization differences to MEG, but large ones to EEG (16.1 ± 4.9 mm), while source orientation differences are non‐negligible to both EEG (14° ± 3.7°) and MEG (12.5° ± 10.9°). Our calibration results show a considerable inter‐subject variability (3.1–14 mS/m) for skull conductivity. The comparison due to different head model show localization differences smaller for EMEG (EW: 3.4 ± 2.4 mm, BT: 3.7 ± 3.4 mm, and PT: 5.9 ± 6.8 mm) than for EEG (EW: 8.6 ± 8.3 mm, BT: 11.8 ± 6.2 mm, and PT: 10.5 ± 5.3 mm), while source orientation differences for EMEG (EW: 15.4° ± 6.3°, BT: 25.7° ± 15.2° and PT: 14° ± 11.5°) and EEG (EW: 14.6° ± 9.5°, BT: 16.3° ± 11.1° and PT: 12.9° ± 8.9°) are in the same range. Our results show that stimulation type, modality and head modeling all have a non‐negligible influence on the source reconstruction of the P20/N20 component. The complementary information of both modalities in EMEG can be exploited on the basis of detailed and individualized head models.

## 1 INTRODUCTION

Electro‐ (EEG) and magnetoencephalography (MEG) source analysis of evoked response components is influenced by a variety of modeling and experimental parameters, some of them are well‐known, while others are often considered to be less important or even negligible (Brette & Destexhe, 2012; Hämäläinen, Hari, Ilmoniemi, Knuutila, & Lounasmaa, 1993). Such parameters are for example the measurement modality, that is, EEG or MEG or combined EEG/MEG (EMEG) and the dielectric properties of the geometrical human head model used for the solution of the forward problem within the inverse source reconstruction procedure (Fuchs et al., 1998; Huang et al., 2007; Vorwerk et al., 2014). Moreover, experimental parameters can vary depending on the interest of the examiner and the complexity of the brain region of interest (Hari & Puce, 2017). Here, we will focus our interest on the human somatosensory system, a well controllable and deeply investigated brain network (Hari & Puce, 2017). Special focus will be on the reconstruction of the somatosensory evoked potential (SEP) and field (SEF) component 20 ms poststimulus, the P20/N20. This is often considered to be largely exogenous and stable with good signal‐to‐noise ratio (SNR) in both EEG and MEG and thus especially appropriate for the sensitivity investigations in this study. According to the sensory “homunculus” (Penfield & Boldrey, 1937), the mainly tangentially‐oriented dipolar source underlying the P20/N20 component was already mapped to Brodmann area 3b on the postcentral wall of the central sulcus in primary somatosensory cortex (SI) contralateral to the side of stimulation (Allison, McCarthy, Wood, & Jones, 1991; Hari & Puce, 2017; Nakamura et al., 1998; Peterson, Schroeder, & Arezzo, 1995). This dipole model is supported by invasive recordings in humans and monkeys (Allison et al., 1991) and by later studies using source analysis of SEP and SEF (Buchner et al., 1994, 1997; Fuchs et al., 1998; Hari et al., 1993; Hari & Forss, 1999; Onishi et al., 2013). However, still unclear is by how much the dipole reconstruction of this component is affected by the fundamental parameters of head modeling, measurement modality, and stimulation type. These sensitivity investigations will be carried out here. In the following, we will first introduce to the three different stimulation types used in our study.

In clinical and research applications, the first transient SI response is typically induced by applying a peripheral somatosensory stimulus. Most often, electric stimulation of the median nerve is applied at the wrist (Allison et al., 1991; Buchner et al., 1994; Fuchs et al., 1998; Hari et al., 1993; Hari & Forss, 1999; Hari & Puce, 2017; Huang et al., 2007; Jung et al., 2003; Kakigi, 1994; Theuvenet et al., 2005). The Electric‐Wrist (EW) stimulation enables high SNR and elicits robust SEP/SEF data with sharp waveforms because a large amount of nerve fibers is synchronously activated by each stimulus and a high stimulation frequency can be applied. However, electric stimulation is rather unnatural and directly activates both deep and superficial receptors, bypassing the peripheral receptors (Nakamura et al., 1998). Additionally, the main drawback of EW stimulation is the discomfort that subjects experience especially after extended periods of stimulation. Therefore, a more physiologically natural excitation using mechanical tactile stimuli has been proposed and applied at the more sensitive (compared to the wrist) fingers (Lew, Wolters, Anwander, Makeig, & MacLeod, 2009; Mertens & Lütkenhöner, 2000; Nakamura et al., 1998; Onishi et al., 2013; Rossini et al., 1996; Simões et al., 2001). One possible technique is to use Pneumato‐Tactile (PT) stimulation using a balloon diaphragm driven by bursts of compressed air. This has been used in several studies showing similar SI responses compared to the electric stimulation, however with a weaker and blurred response (Lew, Wolters, Anwander, et al., 2009; Mertens & Lütkenhöner, 2000; Nakamura et al., 1998). An alternative tactile stimulation type, the so‐called Braille‐Tactile (BT) stimulation, was compared with electric finger stimulation SEF by Onishi et al. (2013), resulting in significantly smaller BT source activation. Here, we will use EW stimulation of the right median nerve as well as BT and PT stimulation of the right index finger. While it seems obvious that these three experimental stimulation types will influence dipole reconstructions, the contribution of the measurement modality (combined EEG/MEG—EMEG or single modality EEG or MEG) and the choice with regard to complexity of the head model are still debated and often considered to be less influential. This motivated us to discuss in this work these sensitivities side‐by‐side. In the following paragraphs, we introduce our modality and head model comparisons.

A very high temporal resolution as offered by MEG and EEG is essential to noninvasively study the cortical SI responses evoked by somatosensory stimuli (Brette & Destexhe, 2012). Previous studies have shown in theory (Dassios, Fokas, & Hadjiloizi, 2007) and in practice (Aydin et al., 2014; Fuchs et al., 1998; Huang et al., 2007) that EMEG exploits the complementary information of both modalities, resulting in source reconstructions that outperform the single modality ones. Here, these three (EMEG, EEG, MEG) modality reconstruction differences will be presented side‐by‐side to the three stimulation type (EW, BT, and PT) and to the head modeling differences, the latter introduced in the following.

Appropriate spatial resolution can be achieved in combination with a suitable head volume conductor model for solving the inverse problem (Brette & Destexhe, 2012; Hämäläinen et al., 1993; Huang et al., 2007; Lucka, Pursiainen, Burger, & Wolters, 2012; Wolters, Beckmann, Rienäcker, & Buchner, 1999), which relies on the realistic simulation of EEG and MEG for a given source in the brain (forward problem). Geometrical and electromagnetic features of the head need to be modeled for an accurate solution of the forward problem. This poses a challenging task due to the high number of differently conductive head tissues and the inter‐ and intra‐individual differences in conductivities of some of the essential head tissues such as the human skull (Haueisen, Ramon, Eiselt, Brauer, & Nowak, 1997; Huang et al., 2007; Vorwerk, Aydin, Wolters, & Butson, 2019). The finite element method (FEM) is a numerical approach for solving the forward problem that offers high flexibility to accurately model the electromagnetic field propagation in such geometrically challenging inhomogeneous and anisotropic head volume conductors (Beltrachini, 2018, 2019; Brette & Destexhe, 2012; Gençer & Acar, 2004; Marin, Guerin, Baillet, Garnero, & Meunier, 1998; Vallaghé & Papadopoulo, 2010; Vorwerk et al., 2014; Wolters et al., 2006). While simpler head models have been used in the above‐mentioned SEP/SEF studies, we will generate more detailed realistic and individually skull‐conductivity‐calibrated FEM head models here.

Simplifications and homogenizations need to be made to the available imaging data, usually magnetic resonance imaging (MRI), for constructing a head volume conductor model. On the one hand, the achievable detailedness of the model depends on the degree to which different tissues can be distinguished in the image data. On the other hand, more precise models require more effort and time for segmentation and more sophisticated mathematical methods and are therefore generally more labor‐intensive and computationally expensive (Windhoff et al., 2011; Vorwerk et al., 2014, 2019; Aydin et al., 2014; Beltrachini, 2018, 2019). Based on MRI data, conventional approaches segment the head into scalp, skull and brain, resulting in a realistically‐shaped three‐compartment isotropic (3CI) head model (Brette & Destexhe, 2012; Fuchs et al., 1998; Huang et al., 2007; Kybic et al., 2005; Stenroos & Nummenmaa, 2016). More detailed approaches segment the brain further into cerebrospinal fluid [CSF], gray [GM] and white matter [WM], and/or the skull into compacta (SC) and spongiosa (SS; Ramon, Schimpf, & Haueisen, 2004; Akalin Acar & Makeig, 2013; Rice et al., 2012; Montes‐Restrepo et al., 2014; Azizollahi, Aarabi, & Wallois, 2016; Cuartas, Acosta‐Medina, Castellanos‐Dominguez, & Mantini, 2019). Moreover, brain anisotropy can be incorporated by using diffusion tensor imaging (DTI) data (Tuch, Wedeen, Dale, George, & Belliveau, 2001; Güllmar, Haueisen, & Reichenbach, 2010; Ruthotto et al., 2011; Cuartas et al., 2019), resulting in six compartment anisotropic (6CA) head models (Aydin et al., 2014; Vorwerk et al., 2014).

As one of the important parameters of the head model, skull conductivity is known to vary inter‐individually and to influence EEG, but not MEG reconstructions (Akalin Acar, Acar, & Makeig, 2016; Azizollahi, Darbas, Diallo, El Badia, & Lohrengel, 2018; Vorwerk et al., 2014, 2019). The importance of individual skull conductivity on EEG and EMEG was emphasized in various studies (Akalin Acar et al., 2016; Aydin et al., 2014; Fernández‐Corazza et al., 2017; Fuchs et al., 1998; Haueisen et al., 1997; Huang et al., 2007; Lew, Wolters, Anwander, et al., 2009; Montes‐Restrepo et al., 2014; Roche‐Labarbe et al., 2008; Vorwerk et al., 2019; Wolters, Lew, MacLeod, & Hämäläinen, 2010). Therefore, in our study and following the recommendations of (Aydin et al., 2014), individual skull conductivity will be estimated in a calibration procedure based on the SEP/SEF data. The MEG is mainly affected by the inner brain compartments (CSF, GM, and WM) close to the sources, while skull and scalp play a minor role (Hämäläinen et al., 1993; Vorwerk et al., 2014). Because of the limited amount of brain structure on the secondary current way from the source to the main sensors, brain anisotropic conductivity might be considered as a less influential parameter (Vorwerk et al., 2014). However, although anisotropy modeling might have a minor effect on the localization of the underlying source, it can significantly affect the source orientation and strength and might therefore be important in especially combined EMEG studies (Cuartas et al., 2019; Güllmar et al., 2010; Vorwerk et al., 2014, 2019; Wolters et al., 2006). The source orientation component is furthermore often considered to also contain important localization information (Rullmann et al., 2009; Salayev et al., 2006). Therefore, in this study, we will use individually skull‐conductivity calibrated anisotropic six compartment head volume conductor models (6CA_Cal) as a reference and compare them with more simplistic head models such as 3CI or 6CA with regard to their P20/N20 dipole reconstructions.

This is the first EEG/MEG source analysis group study for the reconstruction of the somatosensory P20/N20 component investigating the reconstruction sensitivity toward three different experimental conditions (EW, BT, and PT), three different modalities (EEG, MEG, and EMEG) and different individual high resolution 3CI or 6CA head volume conductor models with standard or individually calibrated skull conductivity. We will also investigate the interplay of some of those parameters. We will put all influences side‐by‐side with regard to changes in source location, orientation, and strength within the reconstruction process and thereby answer the following four main questions: How is the source reconstruction of the P20/N20 component affected when changing (a) the modality (EEG, MEG, and EMEG), (b) the detailedness of the head model (3CI or 6CA) for standard and individually calibrated skull conductivity, (c) the stimulation type (EW, BT, or PT) for the most integrated modality (EMEG) and three different head models, and (d) are all parameters similarly influential or can they be ordered by their degree of influence/sensitivity?

## 2 MATERIALS AND METHODS

### 2.1 Participants and procedure

#### 2.1.1 Ethics statement

All subjects signed written consent forms for all measurements that have been approved by the ethics committee of the University of Erlangen, Faculty of Medicine on February 20, 2018 (Ref. No. 4453 B).

#### 2.1.2 Subjects and data acquisitions

Five right‐handed subjects (32 ± 8.8 years of age; 2 females) participated in this study. Somatosensory evoked potentials (SEP) and fields (SEF) were simultaneously acquired in a magnetically shielded room using 80 AgCl sintered ring electrodes (EASYCAP GmbH, Herrsching, Germany, 74 EEG channels plus additional six channels to detect eye movements) and a whole‐head MEG system with 275 axial gradiometers and 29 reference sensors (OMEGA2005, VSM MedTech Ltd., Canada). For the detection of cardiac activity, electrocardiography (ECG) was additionally measured. The MEG reference coils were used to calculate first‐order synthetic gradiometers in order to reduce the interference of magnetic fields originating from distant locations. Prior to the measurements, the electrode positions of the EEG cap were digitized using a Polhemus device (FASTRAK, Polhemus Incorporated, Colchester, VT). Moreover, during the acquisition, the head position inside the MEG was tracked via three head localization coils placed on nasion, left and right distal outer ear canal.

A MAGNETOM Prisma 3.0 T (Release D13, Siemens Medical Solutions, Erlangen, Germany) was used for the acquisition of MRI datasets. We measured a 3D‐T1‐weighted (T1w) fast gradient‐echo pulse sequence (TFE) using water selective excitation to avoid fat shift (TR/TE/FW = 2300/3.51 ms/8°, inversion prepulse with TI = 1.1 s, cubic voxels of 1 mm edge length); 3D‐T2w turbo spin‐echo pulse sequence (TR/TE/FA = 3200/408 ms/90°, cubic voxels, 1 mm edge length) and DTI using an echo‐planar imaging sequence (TR/TE/FA = 9500/79 ms/90°, cubic voxels, 1.89 mm edge length), with one volume with diffusion sensitivity b = 0 s/mm^2^ (i.e., flat diffusion gradient) and 20 volumes with b = 1,000 s/mm^2^ in different directions, equally distributed on a sphere. An additional volume with flat diffusion gradient, but with reversed spatial encoding gradients was scanned and utilized for susceptibility artifact correction (Ruthotto et al., 2011). During T1w‐MRI measurement, gadolinium markers were placed at the same nasion, left and right distal outer ear canal positions for landmark‐based registration of EMEG to MRI. All measurements were done in supine position to reduce head movements and to prevent CSF effects due to a brain shift when combining EEG/MEG and MRI (Rice et al., 2012).

#### 2.1.3 Stimulation and experimental paradigm

For the somatosensory stimulation, we used three different experimental conditions: The first consisted of stimulating the median nerve at the right wrist with monophasic square‐wave electrical pulses having a duration of 0.5 ms. The stimulus strength was increased until a clear movement of the thumb was visible. This type of stimulation is abbreviated as EW stimulation. The remaining two stimulation types used tactile stimulation of the distal phalanx of the right index finger. Pink noise was presented to subjects' ears to mask the acoustic noise caused by both tactile stimulators. The first tactile stimulator was a piezoelectric driven Braille stimulator (Metec GmbH, Stuttgart, Germany), we elevated the central 4 out of 8 individually controllable plastic pins grouped in a 2 × 4 array with a rise‐time of 1 ms. This experimental condition is abbreviated by BT stimulation. Our last type of stimulation, abbreviated by PT stimulation, used a balloon diaphragm driven by bursts of compressed air that was fixed by a plastic spring clip to the right index finger. We compensated for the delay between the electrical trigger and the arrival of the pressure pulse at the balloon diaphragm, as well as the delay caused by the inertia of the pneumatic stimulation device (half‐way displacement of the membrane), which summed up to 52 ms in our measurements.

The data were acquired with a sampling rate of 1,200 Hz and online filtered with a 300 Hz low pass filter. The overall duration of the experiment was 9 min, in which 1,200 trials were measured for EW and PT and 880 trials for BT. The stimulus onset asynchrony (SOA) varied randomly (350–450 ms for EW and PT and 550–650 ms for BT) to avoid habituation and to allow obtaining clear prestimulus intervals for signal‐to‐noise ratio (SNR) determination.

### 2.2 Processing of functional data

The raw EMEG recordings were filtered between 20 and 250 Hz (Buchner et al., 1994) applying a digital bandpass filter including a Hann window in CURRY8.^1^ A notch filter was used to eliminate interferences caused by 50 Hz power line frequency and its harmonics. Subsequently, the preprocessed recordings were separated into equally large segments of 300 ms (100 ms prestimulus and 200 ms poststimulus onset). After visually rejecting the bad channels, reduction of noncerebral activity was performed based on a threshold‐based semiautomatic procedure which is offered in CURRY8 followed by visual inspection of the candidate bad trials in each modality. The SEP/SEF evoked responses were then determined as the average across all trials excluding in average 50 trials per subject and stimulation type.

### 2.3 Processing of image data

T1w and T2w MRI were used to construct individual three‐compartment (3C: scalp, skull, brain) and six‐ compartment (6C: scalp, skull compacta [SC], skull spongiosa [SS], CSF, gray matter [GM], and white matter [WM]) head models. The standard low‐parametric isotropic 3C volume conductor model is still frequently used in source analysis (Brette & Destexhe, 2012). It is therefore instructive to compare the results derived based on 6C and 3C head models. The first module of the automatic segmentation pipeline started with the segmentation of the T1w MRI into scalp, brain (bm_T1), and the three brain tissues CSF, GW and WM using SPM12^2^ embedded in the FieldTrip toolbox (Oostenveld et al., 2011). Subsequently, the T2w MRI was registered to the T1w dataset (T2w_T1wSpace) using a rigid registration approach and mutual information as a cost‐function as implemented in FSL.^3^ T2w_T1wSpace was then used for the segmentation of SC, CSF (CSF_T2) and brain (bm_T2). SS was segmented based on Otsu thresholding (Otsu, 1979) upon the so‐called *masked* T2w_T1wSpace images by the 2 mm eroded SC images (i.e., logic operator *AND* between T2w_T1wSpace and the eroded SC). All the segmented tissues were then imported into a second module for structuring the final segmented model. The first step was to ensure no overlap of GW and WM with the SC mask. Then, CSF was masked (mCSF) using first bm_T2 and CSF_T2 and then GW and WM, also to avoid overlap. Unrealistic holes within the masks were detected and filled using the *imfill* function in MATLAB. A combination of region detection algorithm (*bwconncomp* and *regionprops* in MATLAB) and thresholding was used to avoid unrealistic segmentations after the matching of the masks. The final segmentations were visually inspected to ensure there were no errors. To avoid an unnecessary amount of computational work and without losing accuracy, we cut the model along an axial plane 40 mm below the skull (in average across all subjects), following the recommendations of (Lanfer et al., 2012). Finally, the 3C head model was generated by merging SC and SS into skull and mCSF, GM, and WM into the brain compartment. All steps have been developed in MATLAB using SMP12 via the FieldTrip toolbox, FSL, and MATLAB internal routines and will be made available on demand after publication.

We preprocessed DTI data to reduce eddy current and nonlinear susceptibility artifacts (Ruthotto et al., 2011; Aydin et al., 2014) using FSL and the subroutine HySCO^4^ from the SPM12 toolbox. Diffusion tensors were then calculated and transformed into WM and GM conductivity tensors by an effective medium approach (Rullmann et al., 2009; Tuch et al., 2001). These tensors were later included into the head model to account for WM and GM tissue conductivity anisotropy.

**Figure 1:**
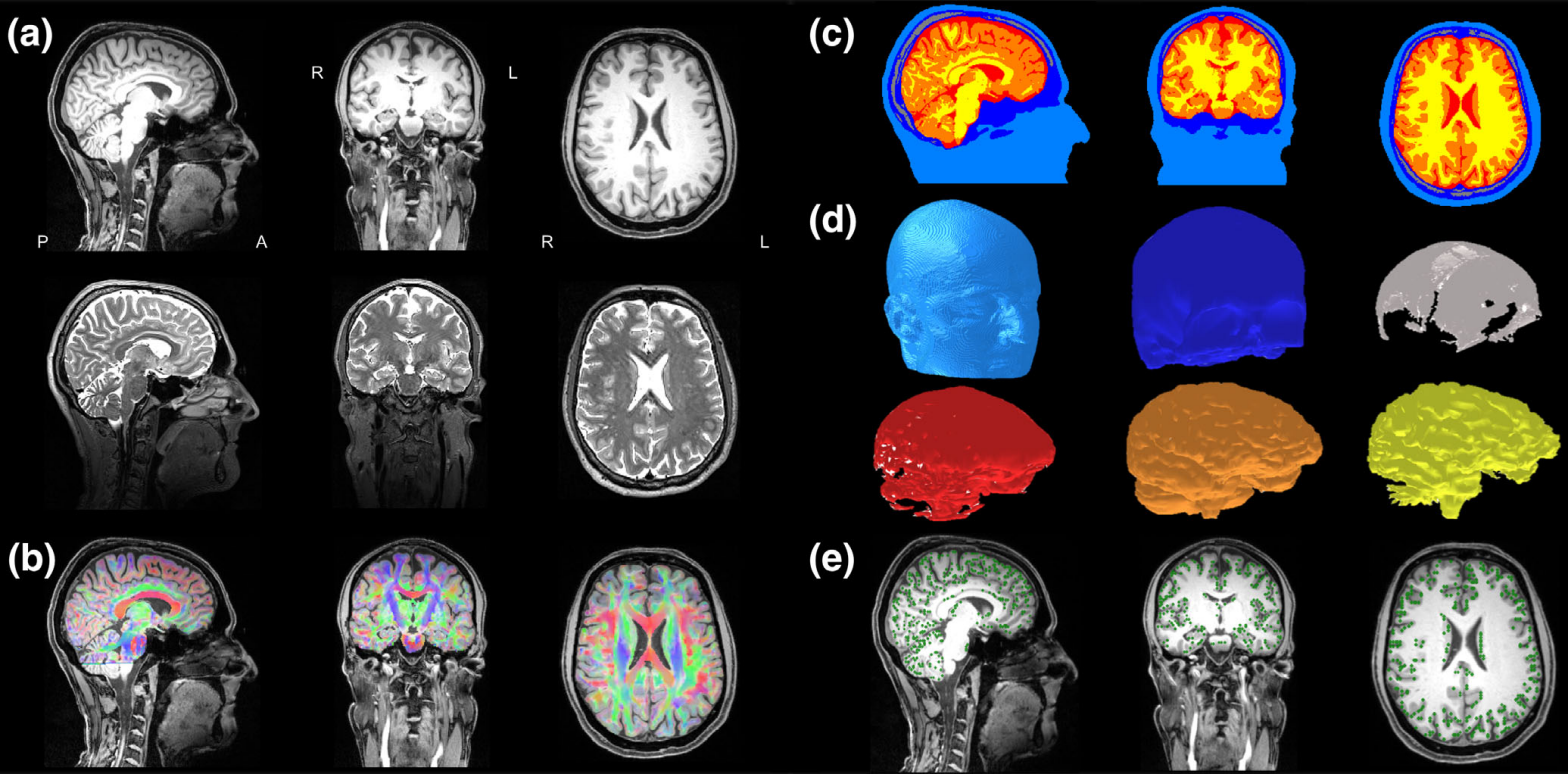
Six‐compartment anisotropic realistic head model and source space: The left subfigure shows sagittal (left column), coronal (middle column), and axial (right column) slices of (a) T1w (upper row) and T2w (middle row) MRIs and (b) a color‐coded (red for left–right; green for anterior–posterior; blue for superior–inferior) fractional anisotropy map computed from the registered DTI and plotted on the T1w‐MRI (lower row). (c) the six‐compartment segmentation result with scalp (light blue), SC (dark blue), skull spongiosa (gray), CSF (red), GM (orange), and WM (yellow; in order to avoid an unnecessary amount of computational work and without losing accuracy, the model in (c) is cut along an axial plane 40 mm below the skull (in average across all subjects), following the recommendations of Lanfer et al. (2012); (d) the geometry‐adapted hexahedral FEM mesh for each of the six tissue compartments (same coloring as in (c)); (e) the source space nodes (green dots) on T1w MRI [Color figure can be viewed at http://wileyonlinelibrary.com]

The resulting segmentation and DTI processing are visualized exemplarily for one of the subjects in Figure 1. Sagittal (left column), coronal (middle column), and axial (right column) slices of T1w (upper row) and T2w (lower low) MRIs are shown in Figure 1a. Figure 1b presents a color‐coded fractional anisotropy map that was computed from the registered DTI and plotted on the T1w‐MRI. Figure 1c shows the six‐compartment segmentation result with scalp (light blue), SC (dark blue), SS (gray), CSF (red), GM (orange), and WM (yellow). In order to avoid an unnecessary amount of computational work and without losing accuracy, we cut the model in Figure 1c along an axial plane 40 mm below the skull (in average across all subjects), following the recommendations of Lanfer et al. (2012).

### 2.4 Calibrated realistic head volume conductor models

In a next step, using the labeled volumes with 1 mm resolution of section 2.3, geometry‐adapted hexahedral finite element (FE) meshes of 1 mm mesh size were constructed for each subject using SimBio‐VGRID^5^ (Wagner et al., 2016; Wolters, Anwander, Berti, & Hartmann, 2007). The adaptation was calculated using a node‐shift of 0.33 ensuring that the interior angles at element vertices were convex and the Jacobian determinant in the FEM computations remained positive. This approach increased the conformance to the real geometries and mitigated the effects of the staircase‐like segmented voxel meshes. The results are three‐compartment isotropic (3CI) head volume conductor models with homogenized skull and brain compartments and six‐compartment head models with brain tissue anisotropy (6CA). The resulting geometry‐adapted hexahedral FEM meshes for each of the six tissue compartments are presented in Figure 1d. In average over the five subjects, these FEM meshes had 3,487,282 nodes and 3,396,950 elements.

#### 2.4.1 Head tissue conductivities

For each subject, we set conductivity values of 0.43 S/m for scalp (Ramon et al., 2004), 1.79 S/m for CSF (Baumann et al., 1995) and 0.33 S/m for the homogenized brain compartment in the 3CI head models (Fuchs et al., 1998; Homma et al., 1995). In the 6CA head models, we used the procedure described in section 2.3 to determine anisotropic conductivity tensors for the compartments GM and WM. While we estimated individual skull conductivity parameters for the 3CI and 6CA models (3CI_Cal and 6CA_Cal, respectively) as described in section 2.4.2 and in section 3, we also generated standard head models with skull conductivity of 0.0041 S/m for 3CI_41 and 0.0041 S/m for the SC and 0.0148 S/m for the SS compartments for the 6CA_41 head models following (Aydin et al., 2014; Buchner et al., 1997; Fuchs et al., 1998; Homma et al., 1995). The value 0.0041 S/m is also implemented as standard skull conductivity in commercial source analysis packages (see, e.g., Fuchs et al., 1998). Thereby, the chosen 6CA_41 skull conductivities furthermore use a fixed ratio for SC:SS of about 1:3.6 (Akhtari, Bryant, & Mamelak, 2002).

#### 2.4.2 Individual skull conductivity calibrations

Here, we have improved a skull conductivity calibration procedure presented in (Aydin et al., 2014, see algorithm 2). It takes into account the different sensitivity profiles of EEG and MEG with specific regard to the insulating skull compartment. In brief: The calibration procedure benefits from the negligible sensitivity of the localization of the P20/N20 SEF component to skull conductivity. We thus used a head model with standard skull conductivity to localize the SEF P20/N20 response, based on the hypothesis that the generator is dipolar, focal, rather lateral and mainly tangentially oriented (Allison et al., 1991; Aydin et al., 2014; Fuchs et al., 1998; Nakamura et al., 1998). In a second step, in order to compensate for the insensitivity of MEG to quasi‐radial source components, we fixed this source location and determined the calibrated individual skull conductivity and the best‐fitting dipole orientation and strength from the corresponding P20/N20 somatosensory evoked potential (SEP) component that overall resulted in lowest residual variance to both EEG and MEG data. We used the EW type for skull conductivity calibration, because of its highest SNR and sharp P20/N20 peak when compared to BT and PT.

While Aydin et al. (2014) used only one conductivity resolution level for calibration, we used a refined version with two resolution levels. We first calculated the calibration optima on the coarser level identically to Aydin et al. (2014), and then further refined them around the individual optima found in level 1. Thereby, our determined skull calibration values enable us to reach an even lower residual variance to the simultaneously measured EW SEP and SEF P20/N20 peaks.

#### 2.4.3 Source spaces

For each subject, a 2 mm resolution source space in the center of the GM compartment without restrictions to source orientations (no normal‐constraint) was constructed. This ensured that all sources were located inside GM and sufficiently far away from the neighboring tissue compartments to fulfill the so‐called *Venant* condition, that is, for each source node, the closest FE node should only belong to elements, which are labeled as GM. It must be fulfilled to avoid numerical problems and unrealistic source modeling for the chosen Venant dipole modeling approach (Vorwerk et al., 2014; Wolters et al., 2007). Figure 1e shows the resulting source space on the T1w MRI in the GM compartment which closely follows the folding of the cortex.

### 2.5 EEG and MEG forward solutions

All forward solutions in this study, including those of section 2.4, were calculated using FEM because of its flexibility with regard to complex geometries and tissue anisotropies (Bauer, Pursiainen, Vorwerk, Köstler, & Wolters, 2015; Beltrachini, 2018; Medani, Lautru, Schwartz, Ren, & Sou, 2015; Pursiainen, Sorrentino, Campi, & Piana, 2011; Vallaghé & Clerc, 2009). For our computations, we used the SimBio^6^ software and the Venant direct source modeling approach (Buchner et al., 1997; Vorwerk et al., 2014; Wolters et al., 2007) due to its high numerical accuracy (Bauer et al., 2015) and high computational efficiency when used in combination with EEG and MEG transfer matrices and an algebraic multigrid preconditioned conjugate gradient (AMG‐CG) solver (Wolters, Grasedyck, & Hackbusch, 2004). Standard piecewise trilinear basis functions were furthermore used in an isoparametric FEM framework (Wolters et al., 2007).

As shown by (Wolters et al., 2004), the used FEM forward computations have linear complexity with the number of nodes when using transfer matrices in combination with the AMG‐CG solver. AMG‐CG was shown to significantly reduce the solution time when compared to simpler solver approaches (Lew, Wolters, Dierkes, Röer, & MacLeod, 2009; Wolters, Kuhn, Anwander, & Reitzinger, 2002) and to be stable towards modeling of tissue inhomogeneity and anisotropy (Wolters, 2003; Wolters et al., 2001). Overall, per subject, the whole skull conductivity calibration process and the leadfield calculations for EEG and MEG for 6CA head modeling needed 15 hr and 15 min on average across all subjects using a standard laptop (Dell, XPS15, 2016), that is, an overnight computation job per subject.

### 2.6 EEG and MEG inverse solutions

Based on the assumption that the generator is focal and single‐dipolar (Allison et al., 1991; Aydin et al., 2014; Fuchs et al., 1998; Hari et al., 1993; Kakigi, 1994; Nakamura et al., 1998), we used single dipole scans (also known as deviation‐, goal function‐, or residual variance‐ scans) to estimate the P20/N20 sources (Fuchs et al., 1998; Knösche, 1997; Wolters et al., 1999) for each measurement modality (EEG, MEG, EMEG). We expected them being located in the primary somatosensory cortex in Brodmann area 3b. Pure MEG dipole scans were regularized in order to suppress the influence of spatially high frequent data noise that might otherwise be amplified into too high radial dipole orientation components (Fuchs et al., 1998; Wolters et al., 1999). No regularization was applied for single modality EEG or combined EMEG source reconstructions. For combined EMEG, since both modalities have different units and residual variance is expressed as the sum of the squared difference over all channels, both need to be transferred to common units. Here we applied the SNR based transformation as suggested by (Fuchs et al., 1998) in order to perform combined EMEG source reconstruction. In this method, the data were whitened according to the noise level (calculated from the prestimulus interval) of each channel so that unit‐less measures for EEG and MEG were obtained to be used in a combined analysis.

## 3 RESULTS

### 3.1 Data analysis pipeline

**Figure 2:**
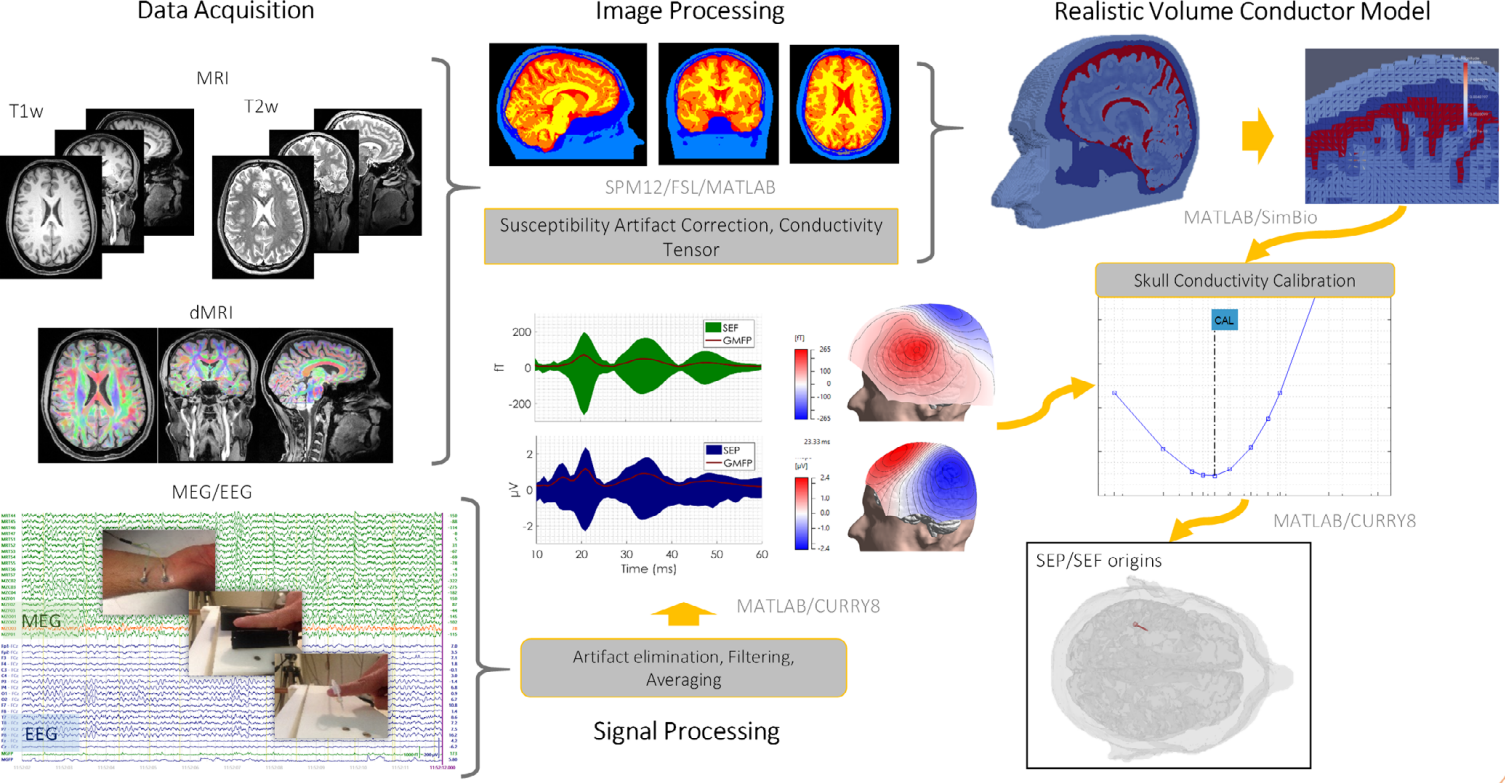
Summary of our data analysis pipeline: The data acquisition block shows the MRI datasets (T1w, T2w, DTI) and the combined SEP/SEF data elicited by electric‐wrist (EW), braille‐tactile (BT), and pneumato‐tactile (PT) somatosensory stimulation. After preprocessing of the structural and functional data, calibrated realistic head models are generated that are then used for source analysis of the somatosensory P20/N20 component. The corresponding software tools used in each step are also indicated [Color figure can be viewed at http://wileyonlinelibrary.com]

Our analysis pipeline is summarized in Figure 2 showing all steps from the raw data up to the source analysis results. T1w, T2w, DTI, and combined SEP/SEF elicited by the three different somatosensory stimulation types (EW, BT, and PT) are shown in the left block (data acquisition). After preprocessing of the structural and functional data, the generated calibrated realistic head models (6CA_Cal and 3CI_Cal) are then used for source analysis of the P20/N20 components.

The remainder of this section is divided into two parts. The first part presents the individual skull conductivity calibration results for the different head models. The second part describes source localization, orientation and strength differences when using different modalities (EEG, MEG, or EMEG), head models (3CI, 6CA, standard, or calibrated) and stimulation types (EW, BT, and PT).

### 3.2 Individual skull conductivity calibration results

**Figure 3:**
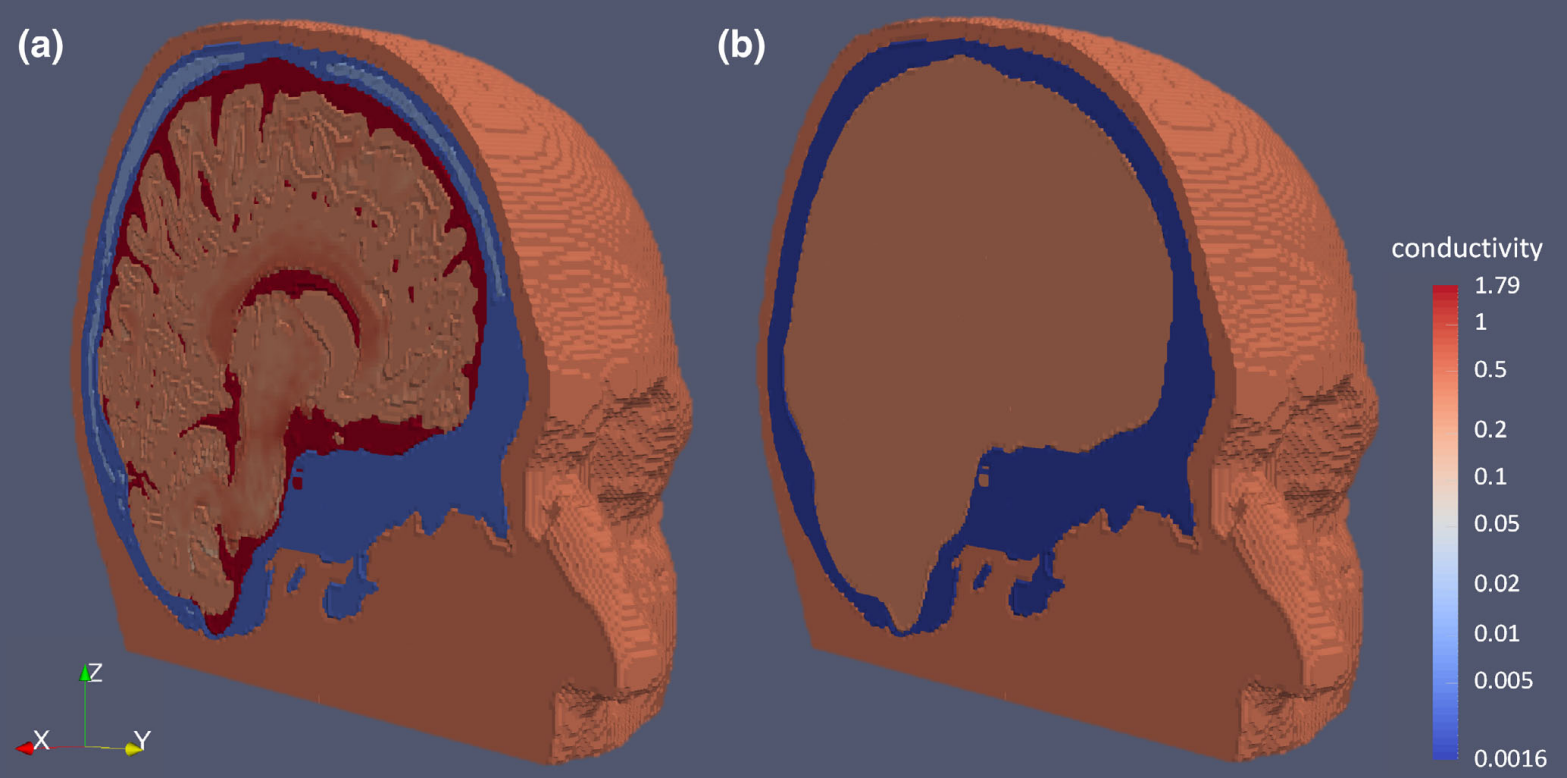
Calibrated head models: (a) Six compartment anisotropic calibrated (6CA_Cal) head model and b) three‐compartment isotropic calibrated (3CI_Cal) head model. Both models are color‐coded (logarithmic scale) according to the conductivity range for subject 1 using a single color‐bar. The spread of the maximum norm of the conductivity tensors, visualized in the brain compartments in model 6CA_Cal, is due to the procedure as described in section 2.4 [Color figure can be viewed at http://wileyonlinelibrary.com]

The calibration procedure described in Section 2.4 was applied to both the 6CA and 3CI head models resulting in their calibrated variants 6CA_Cal and 3CI_Cal, respectively, as shown in Table 1 and Figure 3. For the 6CA head models, calibrated skull conductivities ranged from 0.0031 S/m and 0.0111 S/m (Subject 1, male, age 49) up to 0.014 S/m and 0.0504 S/m (Subject 5, female, age 27) for SC and spongiosa, respectively. For 3CI, calibrated skull conductivities were overall lower, ranging from 0.0016 S/m (Subject 1) up to 0.0083 S/m (Subject 5) for the bulk skull conductivity. Note that the lower values for 3CI_Cal are a result of the interplay between skull conductivity calibration and the modeling differences in the inner compartments, where 3CI uses just a single homogenized brain compartment and 6CA distinguishes CSF, GM, and WM and models tissue anisotropy.

**Table 1:** Overview of the head models for every subject: First column indicates the subject number, the second one shows the gender of the subject (male or female), and the third is about the age (in years)

Subject	Gender	Age	HVCM	Skull	SC	SS
1	M	49	3CI_Cal	0.0016	–	–
			6CA_Cal	–	0.0031	0.0111
2	M	27	3CI_Cal	0.0033	–	–
			6CA_Cal	–	0.0083	0.0299
3	F	27	3CI_Cal	0.0041	–	–
			6CA_Cal	–	0.0045	0.0162
4	M	32	3CI_Cal	0.0033	–	–
			6CA_Cal	–	0.0087	0.0313
5	F	27	3CI_Cal	0.0083	–	–
			6CA_Cal	–	0.014	0.0504

The fourth column indicates the head volume conductor model (HVCM) and the remaining columns the conductivities for the respective compartment resulting from the calibration procedure described in section 2.4.2. The ratio of skull spongiosa to compacta was kept constant to the mean of the ratio measured by (Akhtari et al., 2002) in all 6CA_Cal HVCM. Sign dash was used for the absence of conductivities for nondistinguished compartments in the corresponding HVCM.

6CA_Cal and 3CI_Cal are illustrated exemplarily in Figure 3 to demonstrate the differences in head volume conductor modeling for one of the subjects (Subject 1). Both models are presented using a common color map for the representation of the conductivities. In 6CA_Cal, note the high conductivity of the CSF compartment and the spread of the maximum norm of the conductivity tensors visualized in the GM and WM compartments, which was due to the procedure described in section 2.4. The benefit of our calibration procedure can be appreciated by studying the sensitivity of combined SEP/SEF source analysis to changes in modality (EEG, MEG, and EMEG) or head modeling, as done in the next section.

### 3.3 Effect of modality, head modeling, and stimulation type on the reconstruction of the P20/N20 component

In the following, we tested the sensitivity of the single dipole deviation scans to modality (EMEG, EEG, and MEG) and head modeling (6CA_Cal, 6CA_41, 3CI_Cal, 3CI_41, see Table 1 and Section 2.4). The sensitivity was defined in terms of location or orientation differences and strength reduction between pairs of reconstructed sources. For the location differences, we employed Euclidean distance while we used the normalized inverse cosine of the inner product between the two underlying source for calculating the orientation difference. The strength reduction was calculated by the reduction percentage between the underlying reconstructed sources.

**Figure 4:**
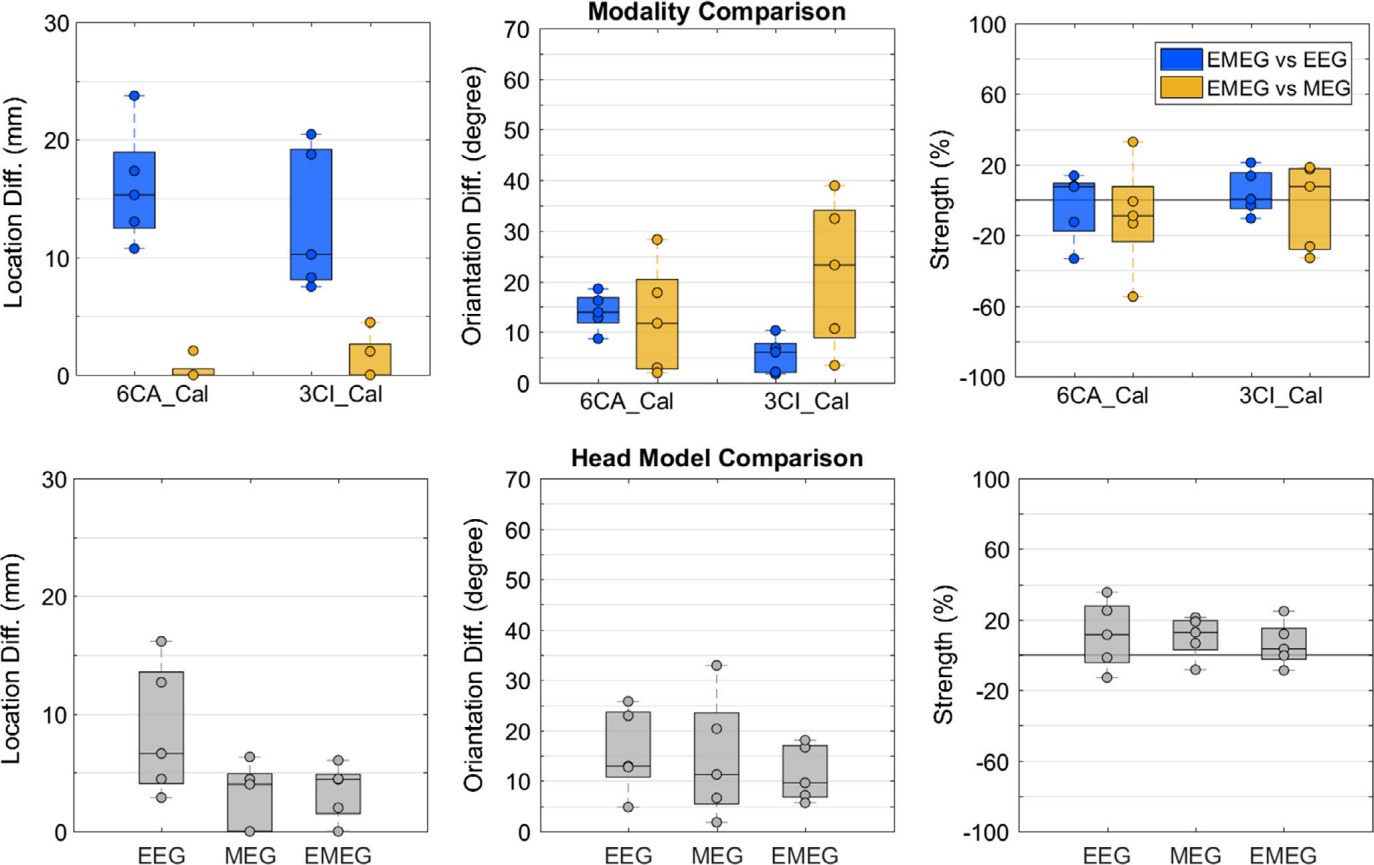
EW (electric‐wrist) stimulation: Effect of modality and head model on P20/N20 reconstruction: Single dipole deviation scan differences for all subjects with regard to source location (left plot, in mm), source orientation (middle plot, in degrees) and source strength (right plot, in %). In (a, upper row), the reconstruction differences are shown when using as a reference the combined EMEG data versus the data of a single (EEG or MEG) modality for the most detailed head model 6CA_Cal (left) and the more homogenized version 3CI_Cal (right) (blue for EMEG vs. EEG and yellow for EMEG vs. MEG). Panel (b) (lower row) depicts the reconstruction differences when using as a reference the most detailed head model 6CA_Cal versus the more homogenized 3CI_Cal for EEG, MEG, and combined EMEG. In each boxplot, the central mark is the median, the edges of the box are the 25th and 75th percentiles. The circles indicate the values for each subject [Color figure can be viewed at http://wileyonlinelibrary.com]

**Figure 5:**
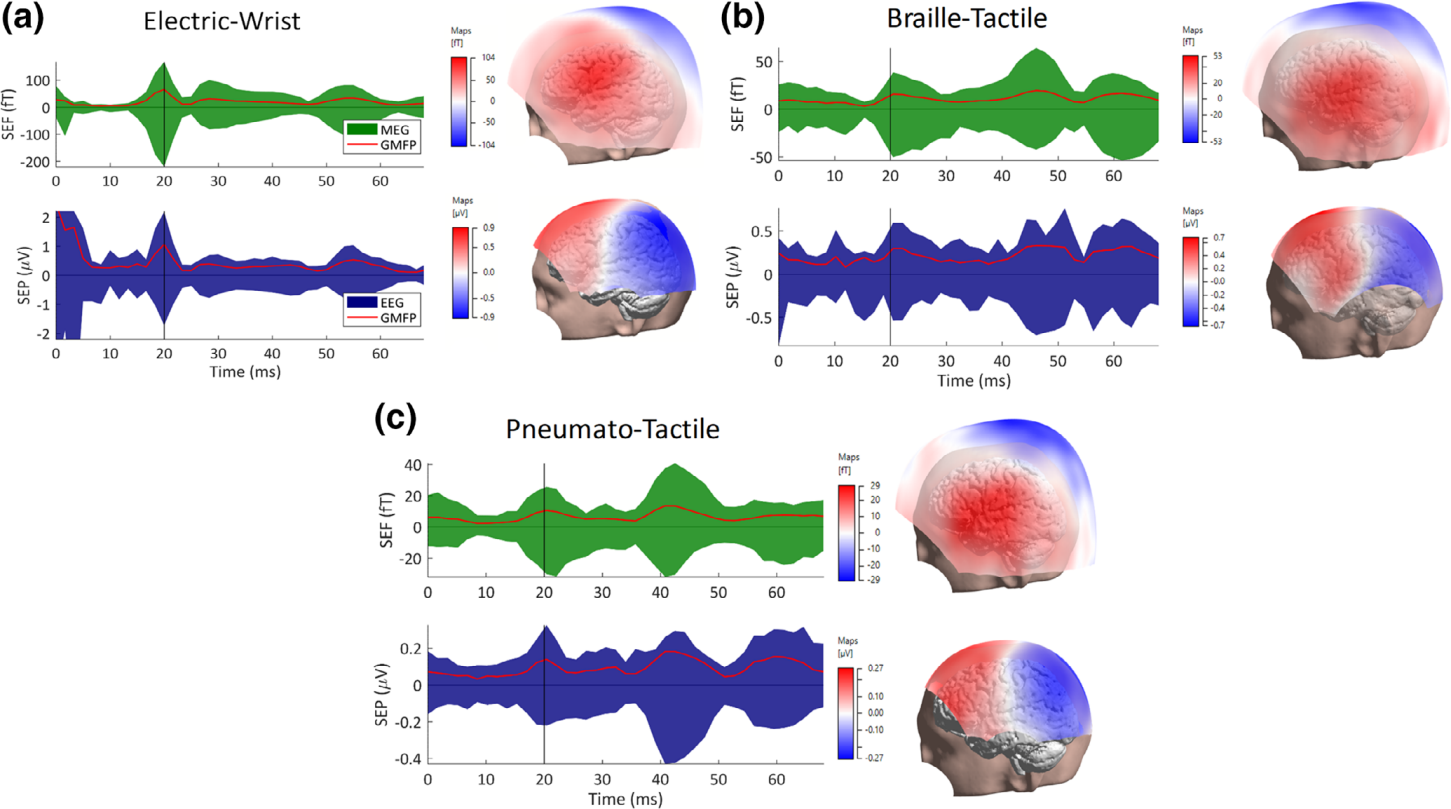
Somatosensory evoked fields (SEFs) and potentials (SEPs) for the three types of stimulation: For the averaged SEFs (upper row) and SEPs (lower row), butterfly plots with global mean field power (GMFP) (left plot, MEG in green, EEG in blue, GMFP in red) and P20/N20 topographies (right plot) are shown from one subject for each type of stimulation: (a) electric‐wrist (EW), (b) braille‐tactile (BT), and (c) pneumato‐tactile (PT). The vertical black line at 20 ms in the left plot represents the highest peak of the P20/N20 component for each stimulation type and the EEG and MEG topographies are then shown in the right plot. Note the different amplitudes for the three stimulation types [Color figure can be viewed at http://wileyonlinelibrary.com]

**Figure 6:**
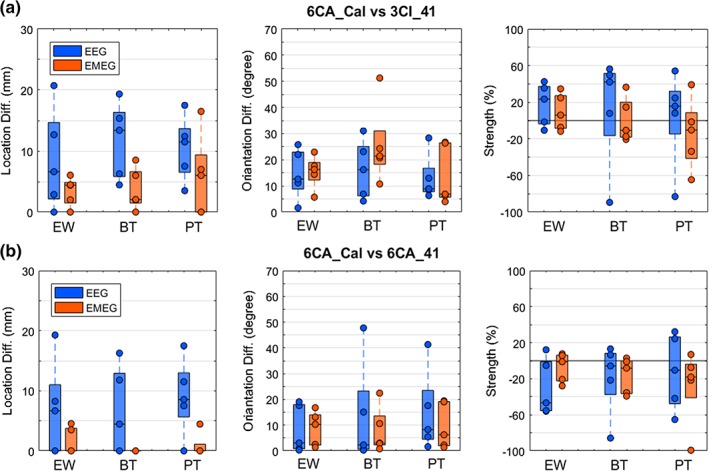
Effect of individual head modeling with focus on skull conductivity calibration on P20/N20 reconstruction: Single dipole deviation scan differences for all subjects with regard to source location (left plot, in mm), source orientation (middle plot, in degrees), and source strength (right plot, in %). Upper row: Most detailed six‐compartment anisotropic head model with individually calibrated skull compartment (6CA_Cal) in comparison to the standard isotropic three‐compartment model (3CI_41). Lower row: 6CA_Cal versus 6CA_41. Results are shown for EEG (blue) and EMEG (orange) and all three stimulation types electric‐wrist (EW), braille‐tactile (BT), and pneumato‐tactile (PT) on the *x*‐axes. In each boxplot, the central mark is the median, the edges of the box are the 25th and 75th percentiles. Notice that the data from the volunteers are overlapping in case that less than five circles are depicted [Color figure can be viewed at http://wileyonlinelibrary.com]

**Figure 7:**
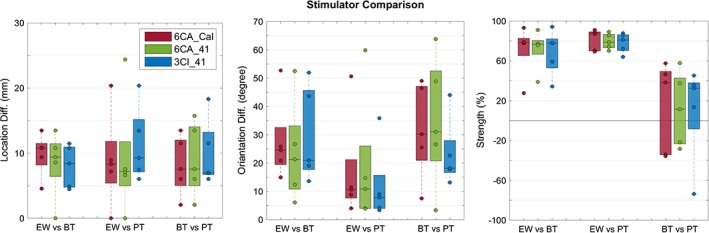
Effect of stimulation type on P20/N20 reconstruction: EMEG single dipole deviation scan differences for all subjects and for the standard head models (3CI_41, in blue), the more detailed head models (6CA_41, in green), and the individually calibrated head models (6CA_Cal, in red) with regard to source location (left plot, in mm), source orientation (middle plot, in degrees), and source strength (right plot, in %). Reconstructions for the stimulation type electric‐wrist (EW) were compared to braille‐tactile (BT), as well as EW to pneumato‐tactile (PT) and BT to PT (*x*‐axes). In each boxplot, the central mark is the median, the edges of the box are the 25th and 75th percentiles [Color figure can be viewed at http://wileyonlinelibrary.com]

Figure 4 depicts the deviation scan differences for all subjects with regard to source location (left plot), source orientation (middle plot) and source strength (right plot). We kept the stimulation type (EW) constant because of the sharper P20/N20 response and the higher SNR when compared to the other two stimulation types (BT, PT; see Figure 5). For easier comparison of the different effects, Figures 4, 6, and 7 have the same maximum *y*‐axis scaling.

At first, we investigated the effect of modality in the source reconstruction of the P20/N20 response. In Figure 4a (upper row), the differences between the source reconstruction of our reference modality (EMEG) and EEG (in blue) and between EMEG and MEG (in yellow) are shown for the most detailed head model 6CA_Cal (left) and the more homogenized version 3CI_Cal (right). As Figure 4a (left plot) shows, with on average 16.1 ± 4.9 and 13 ± 6.1 mm largest localization differences can be observed between EMEG and EEG for 6CA_Cal and 3CI_Cal, respectively (both in blue). The localization results based on EMEG and MEG differed comparably little (<3 mm and <5 mm; 0.41 ± 0.9 and 1.3 ± 1.9 mm) for both calibrated head models (both in yellow). In contrast to this result, for 3CI_Cal, source orientation differences (middle plot) are on average only 5.4° ± 3.6° between EMEG and EEG (blue), but 21.7° ± 14.7° for EMEG versus MEG (yellow). We found larger differences in source orientations for 6CA_Cal having an average of 14° ± 3.7° for EMEG versus EEG (blue), but only 12.5° ± 10.9° for EMEG versus MEG (yellow). Similar source strengths differences (right plot of Figure 4a) can be observed having an average of −3.3 ± 19.5% for 6CA_Cal and 4.3 ± 12.8% for 3CI_Cal for EMEG versus EEG (blue) and −8.96 ± 31.4% for 6CA_Cal and −3.1 ± 24.6% for 3CI_Cal for EMEG versus MEG (yellow). Finally, Figure 4a also depicts a strong inter‐subject variability of the source reconstruction differences that we found for source location, orientation, and strength.

We then examined how the two different calibrated head models (3CI_Cal or 6CA_Cal) affect the source reconstructions of the P20/N20 responses for all three modalities. In Figure 4b, we present these differences between the reconstructions based on the most detailed head model 6CA_Cal in comparison to the more homogenized 3CI_Cal for EEG, MEG, and EMEG. The left plot shows mean source localization differences of 8.5 ± 5.6 mm for EEG, 2.9 ± 2.8 mm for MEG, and 3.4 ± 2.4 mm for EMEG. With regard to source localization, the more detailed head modeling thus mainly influences the EEG modality. However, concerning source orientation (middle plot), larger mean differences between both head models are found for all modalities 15.8° ± 8.5° for EEG, 14.6° ± 12.3 for MEG, and 11.4° ± 5.6° for EMEG. In the right plot of Figure 4b, we found source strength differences between both head models with an average of 11.5 ± 19.5% for EEG, 7.2 ± 11.8% for MEG, and 6.2 ± 12.8% for EMEG. Finally, similar to Figure 4a, also Figure 4b depicts a strong inter‐subject variability of the source reconstruction differences that we found for source location, orientation, and strength.

In the following, we report about the combined SEP/SEF responses for all three stimulation types (EW, BT, and PT, see Figure 5) comparing the effect of the head modeling and modality on the reconstruction of the P20/N20 response within and between the stimulation types (Figures 6 and 7). In the EW dataset (Figure 5a, left column), the components P14 (in EEG), P20/N20 (MEG/EEG) and N30/P30 (MEG/EEG) can be easily recognized due to their high SNR, most prominently the P20/N20, which is of main interest in this investigation. In BT (Figure 5b, left column) and PT stimulation (Figure 5c, left column), the P20/N20 is also well recognizable, but due to the less accurate triggering and, for BT also the lower number of stimuli, SNR is overall poorer. Clear bipolar topographies are illustrated for both modalities (EEG and MEG) and all three stimulation types (EW, BT, and PT) at the peak of the P20/N20 response (Figure 5a–c, right columns). Note the different amplitudes for the three stimulation types in both butterfly and topography plots. Note also that different EEG channels were noise‐corrupted and were therefore deleted in the three stimulation types.

In the upper row of Figure 6, we used the most detailed model 6CA_Cal as a reference and compared the scan result with the one when using the standard isotropic three‐compartment model without skull calibration, namely 3CI_41. For the lower row, the same most‐detailed reference head model 6CA_Cal was compared to a six‐compartment anisotropic head model without skull calibration (6CA_41). Results are shown for all three stimulation types EW, BT, and PT. Because it is well‐known that source localization in MEG is far less sensitive to skull conductivity than in EEG (Hämäläinen et al., 1993; Wolters et al., 2006) and also less affected than EEG with regard to a more detailed modeling of the inner compartments (left plots in Figure 4a,b; Vorwerk et al., 2014), we determined the results shown in Figure 6 only for the modalities EEG and EMEG.

We first used as a reference the most detailed head model 6CA_Cal and compared its reconstructions with those of the standard volume conductor model 3CI_41 (upper row in Figure 6). With mean differences of 8.6 ± 8.3 mm for EW, 11.8 ± 6.2 mm for BT, and 10.5 ± 5.3 mm for PT, EEG source localization (left plot, in blue) was found to be strongly influenced by head modeling. For EMEG source localization (left plot, in orange), smaller mean differences of only 3.4 ± 2.4 mm for EW, 3.7 ± 3.4 mm for BT, and 5.9 ± 6.8 mm for PT are found. As shown in the middle plot, the effect of head modeling on the reconstruction of source orientation is similar for EEG (EMEG) with on average 14.6° ± 9.5° (15.4° ± 6.3°) for EW, 16.3° ± 11.1° (25.7° ± 15.2°) for BT, and 12.9° ± 8.9° (14° ± 11.5°) for PT. Finally, the right plot depicts source strength differences for EEG (EMEG) of on average 17.8 ± 23.1% (9.2 ± 20.1%) for EW, 13.3 ± 60.4% (0.5 ± 24.4%) for BT, and 3.9 ± 51.6% (−14.2 ± 38.5%) for PT.

We then used as a reference to the most‐detailed and calibrated model 6CA_Cal and compared its reconstructions with those of model 6CA_41 with standard skull conductivity (lower row in Figure 6). While with mean localization differences (left plot, in blue) of 6.8 ± 7.9 mm for EW, 6.5 ± 7.3 mm for BT, and 9.0 ± 6.3 mm for PT, SEP reconstructions are strongly affected by skull conductivity variations, combined SEP/SEF differences (left plot, in orange) are rather negligible with 1.6 ± 2.3 mm for EW, no difference for BT and 0.9 ± 2 mm for PT. With EEG (EMEG) source orientation differences (middle plot) of on average 8.2° ± 9.3° (8.8° ± 6.7) for EW, 13.2° ± 20.5° (7.9° ± 8.9°) for BT, and 14.8° ± 15.9° (9.7° ± 8.9°) for PT stimulation and EEG (EMEG) source strengths differences (right plot) of on average − 30.2 ± 31.5% (7.2 ± 16.1%) for EW, −18.6 ± 39.7% (−16.2 ± 19.7%) for BT, and −12.1 ± 41.8% (−27.9 ± 41.4%) for PT stimulation, skull conductivity variations affect source orientations and strengths in both EEG and EMEG. All results in Figure 6 show again a large inter‐subject variability.

In the last Figure 7, the effect of stimulation type on the EMEG deviation scan of the P20/N20 component was investigated for all subjects and for the standard head models (3CI_41, in blue), the more detailed head models (6CA_41, in green), and the individually calibrated head models (6CA_Cal, in red) with regard to source location (left plot), source orientation (middle plot), and source strength (right plot). Reconstructions for the stimulation type EW were compared to BT, as well as EW to PT and BT to PT (*x*‐axes). Source localization differences (left plot) between EW and BT were on average 8.7 ± 3.3 mm, between EW and PT 9.8 ± 7.4 mm, and between BT and PT 9.0 ± 5.0 mm when averaging also over the different head models (because the variation among the different head models was rather small with 3 mm). Large average orientation differences (middle plot) can be noted for EW versus BT (27.1° ± 16.4°) and for BT versus PT (29.90° ± 17.3°), while the orientation differences are on average smaller for EW versus PT (15.9° ± 18.6°). The source orientations in the BT stimulation type thus deviate most from the two other types, regardless of the head model used. This orientation difference can also already be noted in the topography plots in Figure 5. The stimulation type differences between the head models are larger for source orientation (middle plot) than for source localization (left plot). Finally, as shown in the right plot, we find on average 75% higher source strengths for the EW stimulation when compared to BT and PT, and this result is again independent of the head model used. If BT or PT leads to higher source strengths is unclear and differs across individuals (BT vs. PT in the right plot). All results in Figure 7 show again a large inter‐subject variability.

## 4 DISCUSSION

In this sensitivity group study with five healthy subjects, we focused on P20/N20 source analysis for three stimulation types, namely EW stimulation of the right median nerve, BT and PT stimulation of the right index finger. The differences in source reconstructions due to these experimental conditions were compared to the differences due to measurement modality (combined EEG/MEG—EMEG or single modality EEG or MEG) and to the choice of the head model for the solution of the forward problem. We furthermore investigated the effects of forward modeling accuracy on the inverse reconstructions using head models with either the standard three (skin, skull, and brain) isotropic tissue compartments (3CI) or the six (skin, SC, SS, CSF, brain GM and WM) compartments and also brain tissue conductivity anisotropy derived from DTI (6CA). Thereby, our head models used either standard skull conductivity (3CI_41 or 6CA_41) or individually calibrated skull conductivities (3CI_Cal or 6CA_Cal). Our individual‐skull‐conductivity‐calibration results revealed a high inter‐subject variability with values ranging from 0.0031 S/m and 0.0111 S/m up to 0.014 S/m and 0.0504 S/m for SC and spongiosa, respectively. Skull conductivities thus seem to be largely individual, motivating the need for the proposed calibration procedure using simultaneously measured SEP and SEF data. The comparison between pairs of stimulation types showed considerable differences (EW‐BT: 8.7 ± 3.3 mm/27.1° ± 16.4°, BT‐PT: 9 ± 5 mm/29.9° ± 17.3°, and EW‐PT: 9.8 ± 7.4 mm/15.9 ° ± 16.5 ° and 75% strength reduction of BT or PT when compared to EW), regardless of the head model used. EMEG had nearly no localization differences to single modality MEG (0.41 ± 0.9 mm), but large ones to EEG (16.1 ± 4.9 mm), while source orientation differences were non‐negligible to both EEG (14° ± 3.7°) and MEG (12.5° ± 10.9°). When comparing reconstructions in the most detailed and individualized reference head model to the standard three‐compartment head model, localization differences were much smaller for EMEG (EW: 3.4 ± 2.4 mm, BT: 3.7 ± 3.4 mm, and PT: 5.9 ± 6.8 mm) than for single modality EEG (EW: 8.6 ± 8.3 mm, BT: 11.8 ± 6.2 mm, and PT: 10.5 ± 5.3 mm), while source orientation differences for EMEG (EW: 15.4° ± 6.3°, BT: 25.7° ± 15.2°, and PT: 14° ± 11.5°) and EEG (EW: 14.6° ± 9.5°, BT: 16.3° ± 11.1°, and PT: 12.9° ± 8.9°) were in the same range. The standard realistically shaped 3CI head model thus seems to perform reasonably well with regard to source localization when including the MEG modality in EMEG scenarios, but with regard to the source orientation and strength components, it might be too simplistic even for EMEG. Based on our results, it can overall be summarized that stimulation type, modality, and head modeling have a similar and not negligible influence on the source reconstruction of the P20/N20 component. Although the MEG can firmly stabilize the P20/N20 localization, the EEG contributes to the determination of source orientation and strength and the complementary information of both modalities in combined EEG/MEG can be exploited on the basis of detailed and individualized head volume conductor models.

### 4.1 Effects of head modeling and modality on the P20/N20 reconstruction

The individual‐skull‐conductivity‐calibration procedure for 3CI and 6CA revealed lower skull conductivity for the former head model (compare 3CI_Cal to 6CA_Cal in Table 1). This is due to the absence of the highly conductive CSF compartment and the homogenization of the conductivity in the source space area in the realistically shaped 3CI head model (see Figure 3). Similar conductivity sensitivity effects have been found previously using either the FEM or the finite difference method (FDM; Gençer & Acar, 2004; Ramon et al., 2004; Wendel et al., 2008; Vallaghé & Clerc, 2009; Vorwerk et al., 2014, 2019; Azizollahi et al., 2016; Cuartas et al., 2019). Our results, therefore, support the importance of more realistic modeling of these inner brain tissue compartments that also strongly influence than the estimated individual conductivity parameters for the skull layer, as also shown by (Aydin et al., 2014; Fernández‐Corazza et al., 2017). In the current study, we demonstrated this skull conductivity variability by a noninvasive estimation procedure using EMEG source analysis and highly detailed FEM head models in healthy participants.

The head model differences in the forward problems then also had a high impact on the inverse source reconstruction of the P20/N20 responses as shown in Figure 4b for the EW‐type and the comparison 6CA_Cal versus 3CI_Cal and, even more importantly, when comparing reconstructions in 6CA_Cal to the noncalibrated head models 3CI_41 and 6CA_41 in Figure 6. For the standard realistically shaped 3CI head model, even if in Figure 6a the MEG has a stabilizing effect on the EMEG average localization results (EW: 3.4 ± 2.4 mm, BT: 3.7 ± 3.4 mm, and PT: 5.9 ± 6.8 mm) when compared to the much larger single modality EEG differences (EW: 8.6 ± 8.3 mm, BT: 11.8 ± 6.2 mm, and PT: 10.5 ± 5.3 mm), the substantial differences in orientation and strength and the high variability among subjects provides a strong evidence for a considerable influence of the more detailed and individualized head models on the reconstructed activity for both EMEG and EEG. However, largest differences can be seen for the single modality EEG (Figure 6a), confirming EEG simulation results as reported by (Montes‐Restrepo et al., 2014; Akalin Acar et al., 2016; Vorwerk et al., 2019). However, let us now first focus on the localization aspect: The presence of the MEG modality, being less affected by volume conduction effects, is especially important to correctly determine the source depth (Figures 4 and 6a,b and Vorwerk et al., 2019). The source localization difference was only 0.41 ± 0.9 mm between EMEG and MEG (Figure 4a) and both MEG and EMEG localizations were only slightly affected by head modeling differences (Figures 4b and 6). The EMEG localizations were thus mainly driven by the MEG. In accordance to the literature (Aydin et al., 2014; Güllmar et al., 2010), conductivity changes close to the source such as CSF or brain tissue anisotropy affected the source localizations only to a small degree. Hence, about the localizational aspect of source reconstruction, our results are in agreement with the literature and suggest that MEG or EMEG localizations are less affected by volume conduction effects (Aydin et al., 2014; Fuchs et al., 1998; Huang et al., 2007).

Let us now discuss the quality of source reconstruction with regard to the source orientation and strength components, that is, the dipole moments: When using the most detailed and individualized head model 6CA_Cal as the reference, larger source moment differences were found in comparison to 3CI_Cal (Figure 4b, middle and right column) and to 3CI_41 (Figure 6a, middle and right column) and 6CA_41 (Figure 6b, middle and right column), demonstrating the keen sensitivity of source moment reconstructions to the forward modeling accuracy for all three modalities EEG, MEG, and EMEG. A correct reconstruction of the source moment might, however, often be of specific importance. In Salayev et al. (2006), for example, it was suggested that the source orientation component contains important localizational information by predicting the correct (epileptogenic) side of a sulcal wall. Furthermore, in targeted and optimized multi‐channel transcranial electric or magnetic stimulation (TES/TMS), slight changes in target orientation have an even stronger impact on the optimized montages than slight changes in target location (Antonakakis et al., 2019; Dmochowski, Koessler, Norcia, Bikson, & Parra, 2017; Fernández‐Corazza et al., 2017; Schmidt, Wagner, Burger, Rienen, & Wolters, 2015; Wagner et al., 2016). Concerning the impact of head model differences to the reconstructed source moments for the EW stimulation type, EMEG was overall more stable, also with regard to inter‐subject variability, than EEG and MEG alone, as shown for 6CA_Cal versus 3CI_Cal in Figure 4b (middle and right columns) and for 6CA_Cal versus 3CI_41 or 6CA_41 in Figure 6 (left panel in middle and right columns). However, differences between EMEG and the single modalities are only moderate due to the fact that the P20/N20 sources are mainly tangentially oriented, so that even reconstructions from MEG alone are not much more affected than from EMEG (Figure 4b, middle and right columns), at least as long as they are sufficiently regularized (Wolters et al., 1999). Though, the MEG outlier of 33° orientation difference for one subject (Figure 4b, middle column, MEG alone) still points to the fact that source moment reconstructions from MEG alone might be spurious due to the insensitivity of MEG to radial source components. Results for the tactile stimulation types BT and PT are similar to EW as presented in Figure 6 (middle and right columns), except for an overall larger inter‐subject variability, which might in parts also be due to their lower signal‐to‐noise (SNR). Noteworthy is also to note that the differences in source moments presented in Figures 4 and 6 are not only due to changes in head modeling, but they are also resulting from the interplay with the corresponding location differences, that is, more substantial location differences (Figures 4 and 6, left column) most often also result in larger differences in source moments (Figures 4 and 6, middle and right columns). As shown in Figure 4b (right column), the homogenized 3CI_Cal head model resulted for the majority of subjects in a higher source strength, especially for the EEG and to a lesser extent also for the MEG and EMEG modalities. Especially for the EEG and to a lesser degree for EMEG, this is caused by the lower values for calibrated skull conductivity for the 3CI_Cal when compared to the 6CA_Cal head models and by the current channeling due to the additional CSF compartment (Table 1 and Figure 3; see also Vorwerk et al., 2014, 2019; Rice et al., 2012; Güllmar et al., 2006, 2010; Azizollahi et al., 2016; Fernández‐Corazza et al., 2017; Cuartas et al., 2019). Furthermore, in Figure 6 (right column), an increase or decrease of source strength is related to whether the calibrated skull conductivity in Table I is lower or higher than the standard one, resp., further supporting the crucial role of skull conductivity. For the MEG, where skull conductivity does not have this influence, the source strength differences are mainly caused by the interplay with the corresponding localization and orientation differences and, to a lesser degree, by the effects of the additional CSF and brain anisotropy modeling in the more detailed 6CA_Cal head model (Güllmar et al., 2010; Vorwerk et al., 2014).

In summary, it can be observed in Figures 4 and 6 that EMEG reconstructions are less susceptible to forward modeling inaccuracies than single modality EEG or MEG. Combined EEG/MEG exploits the ability of MEG to fairly localize the tangential source components even in the presence of forward modeling inaccuracies, while the EEG contributes its sensitivity to radial (and tangential) source orientation components, being, however, vulnerable to head modeling inaccuracies. Because of this complementarity, the combined analysis of EEG and MEG data is of great interest and might lead to less uncertain source reconstructions and a superior spatial resolution, as has already been shown by others (Baillet, Garnero, Marin, & Hugonin, 1999; Cohen & Cuffin, 1987; Fuchs et al., 1998; Huang et al., 2007; Liu, Dale, & Belliveau, 2002; Lopes da Silva, Wieringa, & Peters, 1991). We demonstrated it here in a group study using three different somatosensory stimulation types and pointed out the importance of using highly detailed and individualized calibrated six‐compartment head models for combined EEG/MEG source analysis.

### 4.2 Effects of stimulation type on the P20/N20 reconstruction

For combined EEG/MEG and when using the most detailed and individualized reference head model 6CA_Cal, localization differences between the stimulation types were in average close to 1 cm (Figure 7, left column, in red: EW‐BT: 8.7 ± 3.3 mm, BT‐PT: 9.0 ± 5.0 mm, and EW‐PT: 9.8 ± 7.4 mm). Our stimulation type localization differences are thus in the same range than those reported for tactile stimulation of either the median nerve or the index finger by (Nakamura et al., 1998). As expected, stimulation type thus has a considerable influence on the P20/N20 localization. We validated our localizations by measuring Euclidean distances of 7–10 mm to the omega‐shaped hand knob area in the motor cortex, showing that the P20/N20 components of all three stimulation types were localized in Brodmann area 3b on the postcentral wall of the central sulcus in primary somatosensory cortex (SI) contralateral to the side of stimulation. This localization result is supported by invasive recordings in humans and monkeys (Allison et al., 1991) and by later studies using source analysis of SEP and SEF (Buchner et al., 1994, 1997; Fuchs et al., 1998; Hari et al., 1993; Hari & Forss, 1999; Nakamura et al., 1998; Onishi et al., 2013). Because simpler head models had been used in these later studies, we also investigated applying the less individualized and/or more homogenized head models 6CA_41 and 3CI_41, resulting in similar localization differences for the three stimulation types (Figure 7, left column, in green and blue, respectively). The stimulation type localization differences can thus also be worked out with simpler head models, even if their absolute localizations differ (Figures 4 and 6). Neurophysiologically, the stimulation type localization differences can easily be explained by the different number of activated neuronal fibers and the resulting different synchronization in SI as well as the resulting different source extent. Furthermore, when considering the differences in source strengths in Figure 7 (right column), it can be expected that the synchronized pyramidal cells in SI of the tactile conditions PT and BT are just different subsets of those of the EW type. Together with the limitations of the focal single dipole model in the localization of slightly extended activated cortical patches (see discussion in De Munck, van Dijk, and Spekreijse (1988)), this then leads to the observed localization differences. However, most importantly, and possibly less expected is our result that the stimulation type localization differences in Figure 7 are in the same range than the differences due to modality and head model, as shown in Figures 4 and 6. It can thus overall be summarized that measurement modality and head modeling play a similarly important role than the less difficult to grasp stimulation type concerning the localization of the P20/N20 component.

We also report distinct differences in source orientation between the stimulation types (Figure 7, middle column, EW‐BT: 27.1° ± 16.4°, BT‐PT: 29.9° ± 17.3°, and EW‐PT: 15.9° ± 16.5°). While the bulk orientation of the pyramidal cells activated by the BT stimulation type differ most from the other two, the source orientation differences between EW and PT are in a similar range than those due to modality (Figure 4a, middle column) or head model (Figures 4b and 6, middle column). The latter shows again that, like for the localizations, modality and head modeling can have a similar effect on source orientation than the stimulation type, where differences are possibly more apparent. For example, in the studies of (Güllmar et al., 2010; Vorwerk et al., 2014, 2019), significant influences of local (i.e., local around the source such as CSF, WM and GM as well as brain anisotropy) and remote (especially skull) conductivity changes on source orientation were shown, in agreement to the differences due to head modeling as presented here. While Onishi et al. (2013) had shown in their SEF source analysis study that depending on the number of activated pins in BT stimulation of the index finger, the resulting localizations within area 3b could easily shift by 5 mm, they did not show the corresponding differences in source orientation, most probably due to the limitations of MEG to the radial source orientation components. However, depending on the local and individual area 3b cortical curvature, a 5 mm shift in localization might easily lead to similar bulk orientation changes than presented in the study at hand. Furthermore, and as already discussed in the last paragraph, source orientation might be specifically important as it can also contain localizational information and as it might be influential in montage optimization for multi‐channel transcranial brain stimulation scenarios. Therefore, we specifically point out here our result that the EW‐PT stimulation type source orientation differences are in the same range than those due to modality and head model. Our comparison study thus gives a better feeling of the contribution that can be expected in specific source analysis scenarios from combined EEG and MEG using detailed individualized realistic head models.

### 4.3 Study limitations and outlook

Because analysis and experimental setup were sophisticated, our study only contained five subjects. However, also with this smaller group size, we could achieve our main result that with regard to source analysis of the somatosensory P20/N20 component, effects of head modeling and modality are in the same range than those of the stimulation type. Further study limitations, which are also reasons for the remaining differences between single modality EEG or MEG and combined EMEG source analysis, are the following: Neither EEG nor MEG data alone allow accurate source analysis of the P20/N20 component. EEG source analysis is sensitive to individual volume conduction parameters, mostly to skull conductivity, but also to a lesser degree to skin conductivity and conductivity of tissues inside the inner skull surface (Aydin et al., 2014; Vorwerk et al., 2019). MEG alone is not sensitive to skull and skin conductivity, but to conductivity of tissues inside the inner skull surface and is especially weak in reconstructing the quasi‐radial source component (Aydin et al., 2014). Skull conductivity calibration, as applied here, should actively alleviate the problem (Vorwerk et al., 2019). Therefore, our results support the notion that the detailed head models and the additional computational costs (per subject, the calibration process and the calculation of all lead fields were performed in an overnight computation job) seem justified by the possible gain in precision. However, a remaining difference between EEG, MEG, and EMEG source analysis, as also observed in our results, has to be expected even with the detailed and individualized head models used here. Thus, even though great care has been taken to construct subject‐specific individualized multi‐compartment realistic head models, our models still contain simplifications, which might result in modeling errors. For example, in a recent meta‐analysis of reported human head electrical conductivity values, (McCann, Pisano, & Beltrachini, 2019) did not only report variations in skull conductivity but also in other conductivity parameters, for example, scalp conductivity. In (Arumugam, Morgan, Kim, Kuo, & Turovets, 2018; Fernández‐Corazza et al., 2017), also other individual parameters such as scalp conductivity were determined using Electrical Impedance Tomography (EIT) techniques. In our study, we individualized skull and brain conductivity but kept other conductivity parameters fixed at their standard constant value. Furthermore, in Aydin et al. (2017) and Ramon, Garguilo, Fridgeirsson, and Haueisen, (2014), it was shown that the dura might also play a vital role, at least for EEG forward modeling. Dura has not been modeled explicitly in our head models, only implicitly by influencing our bulk calibration values. Furthermore, our Polhemus‐procedure for EEG sensor registration, the fiducial‐based registration of EEG and MEG onto the MRI, as well as subject movements in EEG/MEG and in MRI might cause artifacts that are also reflected in persisting differences between EEG, MEG, and EMEG reconstructions.

As an outlook, based on the results of this study and on the first clinical success in presurgical epilepsy diagnosis (Aydin et al., 2015, 2017), we plan to further push forward combined EEG/MEG source analysis using detailed individualized realistic head models. To do so, our new software framework *Duneuro* (Nüßing, Wolters, Brinck, & Engwer, 2016; Engwer et al., 2007; Piastra et al., 2018) will enable even more accurate individualization and calibration in a more automatic way. Currently, we still recommend calibration based on EW stimulation. However, in order to investigate if the more comfortable BT stimulation might replace this standard, we have already investigated SNR, stimulator artifact and SEF source analysis differences due to different number of pins in BT stimulation (data not shown). These results motivated us to use the middle four out of eight pins that our BT stimulator offers. We now plan to investigate the influence of different pin numbers and patterns also in EEG and EMEG source analysis scenarios as well as the impact of the stimulation type on our calibration procedure.

## 5 CONCLUSION

In this sensitivity group study of five healthy participants, we showed that different stimulation types, measurement modalities, and head modeling procedures all have a similar and non‐negligible influence on source analysis of the somatosensory P20/N20 component. Our study thus makes it possible to record the effect sizes of modality and head modeling in comparison to the more easily understandable effect size of stimulation type. Further results are that a high inter‐individual skull conductivity variability was observed throughout the healthy participants by our non‐invasive calibration procedure using EW SEP and SEF. Using three different somatosensory stimulation types, besides EW also BT and PT, we demonstrated that combined EEG/MEG (EMEG) source reconstructions are less susceptible to forward modeling inaccuracies than single modality EEG or MEG and showed the importance of using highly detailed and individualized calibrated six‐compartment FEM head models. While the MEG is the superior modality for P20/N20 localization, both modalities contribute to the determination of source orientation (MEG only with regard to the tangential source orientation component) and the estimation of individual skull conductivity and the complementary information of both modalities in combined EMEG can be exploited on the basis of detailed and individualized head volume conductor models. The combined analysis of EEG and MEG in combination with detailed head modeling is thus a promising approach that might lead to more stable source reconstructions even in the presence of unavoidable remaining modeling errors, and thus to an overall superior spatial resolution.

## CONFLICT OF INTEREST

None declared.

## Data Availability

The data that support the findings of this study are available on request from the corresponding author. The data are not publicly available due to privacy or ethical restrictions.

## Footnotes

1
https://compumedicsneuroscan.com/products/by-name/curry/

2
http://www.fil.ion.ucl.ac.uk/spm/software/spm12/

3
http://www.fmrib.ox.ac.uk/fsl

4
http://www.diffusiontools.com/documentation/hysco.html

5
http://vgrid.simbio.de/

6 SimBio: https://www.mrt.uni-jena.de/simbio/index.php/Main_Page and its integration into FieldTrip (see Vorwerk, Oostenveld, Piastra, Magyari, & Wolters, 2018)
